# Polylactide (PLA) as a Cell Carrier in Mesophilic Anaerobic Digestion—A New Strategy in the Management of PLA

**DOI:** 10.3390/ma15228113

**Published:** 2022-11-16

**Authors:** Agnieszka A. Pilarska, Karol Bula, Krzysztof Pilarski, Mariusz Adamski, Agnieszka Wolna-Maruwka, Tomasz Kałuża, Przemysław Magda, Piotr Boniecki

**Affiliations:** 1 Department of Hydraulic and Sanitary Engineering, Poznań University of Life Sciences, Piątkowska 94A, 60-649 Poznan, Poland; 2 Institute of Materials Technology, Faculty of Mechanical Engineering, Poznan University of Technology, 60-965 Poznan, Poland; 3 Department of Biosystems Engineering, Poznań University of Life Sciences, Wojska Polskiego 50, 60-627 Poznan, Poland; 4 Department of Soil Science and Microbiology, Poznań University of Life Sciences, Szydłowska 50, 60-656 Poznan, Poland; 5 Department of Wastewater Treatment, Aquanet S.A., Gdyńska 1, 61-477 Poznań, Poland

**Keywords:** polylactide, cell carrier, thermal properties, bacterial proliferation, anaerobic digestion, process efficiency

## Abstract

The management of waste polylactide (PLA) in various solutions of thermophilic anaerobic digestion (AD) is problematic and often uneconomical. This paper proposes a different approach to the use of PLA in mesophilic AD, used more commonly on the industrial scale, which consists of assigning the function of a microbial carrier to the biopolymer. The study involved the testing of waste wafers and waste wafers and cheese in a co-substrate system, combined with digested sewage sludge. The experiment was conducted on a laboratory scale, in a batch bioreactor mode. They were used as test samples and as samples with the addition of a carrier: WF—control and WFC—control; WF + PLA and WFC + PLA. The main objective of the study was to verify the impact of PLA in the granular (PLAG) and powder (PLAP) forms on the stability and efficiency of the process. The results of the analysis of physicochemical properties of the carriers, including the critical thermal analysis by differential scanning calorimetry (DSC), as well as the amount of cellular biomass of *Bacillus amyloliquefaciens* obtained in a culture with the addition of the tested PLAG and PLAP, confirmed that PLA can be an effective cell carrier in mesophilic AD. The addition of PLAG produced better results for bacterial proliferation than the addition of powdered PLA. The highest level of dehydrogenase activity was maintained in the WFC + PLAG system. An increase in the volume of the methane produced for the samples digested with the PLA granules carrier was registered in the study. It went up by c.a. 26% for WF, from 356.11 m^3^ Mg^−1^ VS (WF—control) to 448.84 m^3^ Mg^−1^ VS (WF + PLAG), and for WFC, from 413.46 m^3^ Mg^−1^ VS, (WFC—control) to 519.98 m^3^ Mg^−1^ VS (WFC + PLAG).

## 1. Introduction

The generation of energy from non-renewable sources is currently considered one of the most important causes of the climate crisis related to global warming. The highest emissions of methane, which is one of the most important greenhouse gases, accompany, to a large extent, the extraction of fossil fuels [1], the management of waste and wastewater, the decomposition of manure, and the combustion of biomass and biofuels. Anaerobic digestion (AD) is one of the most practical approaches constituting an alternative energy source to fossil fuel extraction [2,3]. It is also a well-tested strategy for managing agricultural and food waste. The possibility of using organic waste in the AD process offers a wide range of possibilities from economic and environmental points of view. On the other hand, it poses limitations related to the need for optimisation activities and the search for technologies to improve substrate bioconversion [4,5]. Innovative biotechnology solutions provide key support in this respect.

The good condition of the bacterial flora as a catalyst for biochemical transformations is a factor that makes it possible to obtain high calorific biogas released in the AD process, as well as nutrient rich digestate [6,7]. One of the methods of improving the conditions for the functioning of methanogens is immobilising them with an appropriate carrier. Interactions between the microorganisms and the support material lead to an efficient formation of a biofilm, the durability of which depends on, i.e., the type of the matrix and individual characteristics of the environment [8]. The available research reports contain information about the implementation of AD experiments involving, i.e., zeolites, bentonite, perlite, activated carbon, natural rubber, chitosan, lignin, or silica [9,10]. However, the aforementioned materials, in many cases, are affected by functional imperfections such as low surface area and limited mechanical strength, due to which they are not always sufficiently effective. Recently, special attention has also been given to conductive materials, such as biocarbon, carbon nanotubes, and magnetite, which are applied under the conditions of anaerobic biodegradation, with the view to improving the efficiency of methanogenesis through the direct interspecies electron transfer (DIET) [11,12]. However, the knowledge of processes of cell activation and development in the presence of innovative matrixes continues to have some gaps [13] and, therefore, requires further exploration and continual attention from researchers.

Given that cell carriers in biotechnological applications, including in AD, should be stable under process conditions and environmentally friendly, polylactide (or poly(lactic acid), PLA) has been identified as the current leader in the market of biopolymers. PLA is a biocompatible, thermoplastic, aliphatic polyester produced, i.e., from lactic acid, which is manufactured biotechnologically with the use of renewable natural resources such as corn starch and sugar cane [14]. Although this polymer has been known for a long time, it was only in the new century that its production and processing on an industrial scale were developed to a significant extent. The use of post-production and post-consumption waste with PLA as a microbial additive improving the performance of specific bacteria strains in the AD process can be an excellent alternative for the management of that waste.

So far, PLA has been most frequently used in medicine, chemistry, the pharmaceutical industry, and in eco-friendly industrial production as a promising product associated with the concept of “green plastic” [15,16]. The use of PLA as temporary extracellular matrices in tissue engineering should be highlighted in the first place. On the other end of the spectrum are PLA’s applications as drug-loaded carriers, polymeric nanoparticles, dendrimers, and micelles [15]. Thanks to the development of production technology for PLA and its composites with defined dispersion and thermo-mechanical properties [17,18,19], it is possible to obtain materials with properties resembling conventional plastics. Currently, the progress has resulted in a wide range of applications for PLA, including in areas such as the packaging industry, textiles, agriculture, construction, and others [14,20]. 

Mechanical and thermal properties provide PLA with good functional characteristics, also considering the use of this biopolymer in biotechnological processes, i.e., as a carrier in the AD process. In terms of mechanical properties, PLA displays a high tensile modulus (3 GPa) and high yield strength (50–70 MPa). PLA has a glass transition (*T*_g_) value around 60 °C [19]. This characteristic point refers to a change in the mobility of amorphous chains [21,22]. Isotactic polylactides crystallise forming a homocrystal, which melts in the range of 160–190 °C depending on the molecular mass [23]. PLA is a biodegradable polymer but, importantly, only under strictly defined conditions, which include temperature increased to at least 58 °C, access to moisture, and minimum time, which amounts to six months (depending on the condition of the medium). Moreover, PLA is a material that is highly soluble in 1,2 dichloroethane, toluene, and tetrahydrofuran but is insoluble in water and acetone, and in some alcohols and alkanes [24].

The research on the use of PLA as a substrate in anaerobic digestion conducted so far has focused on its complete biodegradation. The key research problem in the conducted experiments consisted of the selection of appropriate conditions, i.e., temperature and pH of the process environment. As a result, the research work that was carried out demonstrated that the decomposition of PLA in the AD process is difficult and fundamentally impossible to achieve under mesophilic conditions. According to Cazaudohore et al. (2022) [25], such polymers, including, for example, poly(butylene succinate), appear to be biodegradable in the conditions of industrial composting but not during digestion, even when it lasts a long time. Currently, little is known about microorganisms involved in the anaerobic digestion of biodegradable plastics. It is believed that there is no significant population of microorganisms existing under anaerobic conditions which would directly degrade semicrystalline PLA with a high molecular mass [26,27]. Authors of numerous research papers, attempting to develop effective technology for PLA decomposition in the AD process, have mostly applied the following: significantly elevated temperatures, i.e., conditions of a hyperthermophilic (70–80 °C) and thermophilic (55 °C) reactor [28], pre-treatment under alkaline conditions [29,30], and co-digestion [31]. It should be remembered, however, that the decisive majority of biogas plants operate under mesophilic conditions due to economic reasons and due to destabilisation problems caused by the high temperature required by the process. The use of commercial additives increases the costs of the process. Meanwhile, the composting of biopolymers in biologically active landfills as a disposal method involves a threat of releasing emissions into the atmosphere and needs to be perfected [26]. Therefore, it is justified to involve PLA in mesophilic digestion as a cell carrier that is relatively durable under these conditions and potentially capable of improving the efficiency of the AD process. The biopolymer remaining in the digestate, decomposed to lactic acid, will acidify the soil, protecting it against pathogenic bacteria and serving as a source of carbon for soil microorganisms [32]. This strategy can support the management of PLA and partially solve the existing research and scientific problems.

The purpose of the study was to assess the potential for the use of PLA in the form of granules (PLAG) and powder (PLAP) as a cell carrier in mesophilic AD on the laboratory scale, using food waste as a substrate. An analysis of the physicochemical and thermal properties of PLA was carried out, and the cumulative biogas/methane yield for control samples and carrier samples was determined. The enzymatic activity of the digest samples and parameters of the process stability were examined during the process. Cell proliferation in the PLA carrier environment was also assessed on the basis of the obtained cell biomass.

## 2. Materials and Methods

### 2.1. Feedstock and Inoculum Sources

The raw materials for biogas production used in this study included food waste from a confectionery plant (waste wafers with filling, WF) and from a dairy plant (waste cottage cheese (CHE)). Both plants were located in the vicinity of Poznań. As in the previous experiments, digested sewage sludge (SS) supplied by the municipal sewage treatment plant in Poznań was used as inoculum. At the same time, it also acted as an effective buffer for the system [33]. 

Table 1 presents the values of physicochemical parameters of the substrate and inoculum used in the discussed experiment.

### 2.2. Carrier Source and Preparation 

The carrier used in the experiment was PLA, a biopolymer manufactured by Nature Works LLC, Plymouth, USA with its grade name Ingeo 2500HP in the form of granules and in a powder state. The powder was manufactured through pulverising with the use of granules. To prepare a small fraction of pulverised PLA 2500HP, an adequate amount of polylactide granules was fed into the ultra-centrifugal mill Retsch RM 200 (Retsch GmbH, Haan, Germany). The centrifugal disc was operated at a speed of 6000 rpm which allowed for a very short residence time of granules in contact with metallic elements. Rotating disc using centrifugal force threw pellets into the razor screen with opening size of 2 mm. Additionally, an automated vibratory feeder (DR 100 Retsch (Retsch GmbH, Haan, Germany)) was used for maintaining the appropriate dosage. By using automatic feeding, a uniform amount of material was directed into the mill chamber, which prevents overheating of the material and ensures standardised residence time in the grinding chamber.

### 2.3. Anaerobic Digestion

#### 2.3.1. Batch Preparation 

So-called control inputs were prepared to carry out the experiment. They were prepared on the basis of sewage sludge, which served as a bacterial inoculum, waste wafers (WF–control), and waste wafer and cheese system (WFC–control), which served as a comparison for the tested samples with the addition of PLA in the form of granules (WF/WFC + PLAG, G—granules, pellets, WF/WFC + PLAP, P—powder). The ratio between the substrate and the inoculum in the inputs was determined on the basis of a German standard VDI 4630 concerning the digestion of organic materials, characterisation of substrates, sampling, collection of material data, as well as digestion tests [34]. As recommended, the content of dry organic matter in the inoculum ranged from 1.5% to 2%, whereas the content of dry matter in the batch did not exceed 10%. The low content of total solids in the sludge (Table 1) and their domination in the samples (Table 2) ensured that the mixture could be easily pumped in the technical plant at the temperature at which the process was carried out. The qualitative and quantitative composition of the SS was subject to change, while the high content of organic components made it unstable. The results of SS tests point to the significant influence of weight concentration, temperature, specific surface area, and effects of digestion on their rheological parameter values [35]. The rheology of SS is an important issue used in the work on the methods of management and control of sewage sludge in the processes of dehydration and stabilisation [36].

#### 2.3.2. Biogas Production and Analysis

AD was conducted in a multi-chamber batch bioreactor, the structure of which was outlined and discussed in detail in the author’s earlier publications [33,37]. The process was conducted in mesophilic conditions (38 °C). The total number of digestion tanks (with a capacity of 1.0 L) involved in this study amounted to 18 (each sample was tested three times). The batches were mixed once a day. Mixing improves the efficiency of the process, which is achieved, i.e., by means of maintaining an even distribution of heat and nutrients, preventing local rotting processes, and the formation of foam and bottom deposits. 

The hydraulic retention time (HRT) in the process amounted to 21 days. According to the German standard DIN Guideline 38 414-S8 (DIN, Deutsches Institut für Normung) [38], the experiment continued until the daily biogas production fell below 1% of the total amount of biogas produced in all biodigesters. The amount of produced biogas was checked every 24 h. The concentration of methane, carbon dioxide, hydrogen sulphide, ammonia, and oxygen in the biogas was measured with the Geotech GA5000 gas instrument (manufactured by Geotech, Coventry, UK). The gas concentration was measured with Mg-72 and Mg-73 measurement devices from Alter inc., Tarnowo Podgórne, Poland. The gas analyser measures gas concentrations within the following ranges: 0–100% CH_4_, 0–100% CO_2_, 0–25% O_2_, 0–2000 ppm H_2_S, and 0–1000 ppm NH_3_. The assessment of biogas yield (in m^3^ Mg^−1^), in terms of total solids and volatile solids, was performed on the basis of experimental data. The cumulative amount of biogas, including methane, obtained from individual samples, was calculated on the basis of formulas presented in the authors’ earlier work [33,37]. 

### 2.4. Analysis of Substrate and Digestate Samples

The substrates and the prepared inputs were subject to pH measurement (potentiometric analysis) with Elmetron CP-215 (ELMETRON, Zabrze, Poland) instrument. For the same material, total solids (TS) were also measured by drying at 105 °C (Zalmed SML dryer, Zalmed, Łomianki, Poland) while volatile solids (VS) were measured by combustion at 550 °C (MS Spectrum PAF 110/6 furnace, MS Spectrum, Warsaw, Poland)—gravimetric analysis. The methodology for determining other physicochemical parameters of the substrates was outlined in the author’s earlier publications [3].

The digested samples were also tested for nitrogen content, with the titration, Kjeldahl method using 0.1 n HCl in the presence of the Tashiro’s indicator, and for ammonium nitrogen content, with the distillation and titration method using 0.1 n HCl, in the presence of the Tashiro’s indicator. To determine the concentration of VFA (volatile fatty acids), TA (total alkalinity), and then the VFA/TA ratio (volatile fatty acids-to-total alkalinity ratio) in the digested batches, 5 mL of a given sample was collected, then titrated by 0.1 N of sulphuric acid solution (H_2_SO_4_) up to pH 5.0 to calculate the TA value. The VFA value was obtained after a second titration step between pH 5.0 and pH 4.4 [5]. 

### 2.5. Analysis of Carrier’s Properties

#### 2.5.1. Microscopy and Microstructural Investigations 

For the evaluation of the granulometry and assessment of the particle shape of pulverised PLA particles, an optical stereo microscope Opta-Tech SK with fibre optic spot was utilised (Opta-Tech, Warsaw, Poland). The magnification of the viewing head applied ranged from 7× to 40×.

The morphology and microstructure of the PLA were examined on the basis of SEM (scanning electron microscope) images recorded from a scanning electron microscope FEI Quanta FEG 250 (FEI Company, Hillsboro, OR, USA). The microscope operates in low vacuum mode at 70 Pa using an accelerating voltage of 10 kV. Before testing, the samples were coated with Au for a period of 5 s using a Balzers PV205P coater (Balzers, Switzerland) [39]. The EDS (energy dispersive X-ray spectroscopy) analysis was carried out with the beam accelerating voltage of 10 kV, using the EDS Octane SDD (EDAX Inc., Mahwah, NJ, USA) detector. The contents of elements such as C, O, and Si were analysed. The distribution of element concentrations in the form of EDS pattern maps was performed.

#### 2.5.2. Apparent Density of PLA Granules and Powder State

The determination of the apparent density of bulk materials was characterised using PI-MGES (Polon-Izot, Warsaw, Poland) apparatus that uses a specified funnel and a small container with 100 mL capacity, according to EN ISO 60:2000 standard [40]. The sample was poured through a specified funnel into a measuring cylinder of 100 cubic centimetre capacity, the excess was removed with a straightedge, and the mass of the contents was determined by weighing. The apparent density was expressed in grams per millilitre. The mean value of the measured density was calculated from each of the three individual tests.

#### 2.5.3. Elemental Analysis

The measurements were made with the FLASH 2000 CHNS/O Organic Elemental Analysis analyser (Thermo Scientific, Waltham, MA, USA). Flash 2000 analyser relies on dynamic combustion technology. In the first stage, samples were weighed in tin capsules (approx. 2–4 mg) and placed in the reactor with an autosampler with a specific amount of oxygen. After combustion at 900–1000 °C combustion gases were transported in a helium flow to the second reactor furnace filled with copper and then moved via a water trap to the chromatography column. Eventually, the separated gases were detected by thermal conductivity detector (TCD). The Thermo Scientific Eager Xperience data analysis software automatically generates and displays a report at the end of the measurement cycle [41]. Meanwhile, oxygen content analysis was performed by the instrument in the pyrolysis mode. Samples were weighed in silver capsules and placed in the furnace with an autosampler. The reactor contained nickel-coated carbon and its temperature was maintained at 1060 °C. The oxygen in the sample combined with carbon to form carbon monoxide, which was subsequently chromatographically separated from the other products and measured by a TCD detector.

#### 2.5.4. Differential Scanning Calorimetry Analysis

Differential scanning calorimetry (DSC) measurements were taken on the Netzsch DSC 204 F1 Phoenix (Selb, Germany) calorimeter. The samples (each ca. 10 mg) were heated up to 230 °C at a rate of 10 °C /min under a nitrogen atmosphere and maintained at this temperature for 5 min in order to eliminate the thermal history that influenced the first heating step. In the second step, the samples were cooled from 230 to 20 °C at a cooling rate of 20 °C /min. This procedure was repeated two times, and the second segment was provided to calculate the crystallinity degree (*X_c_*) from the melting enthalpy. The enthalpy melting of 100% crystalline PLA matrix was defined as 93.0 J g^−1^, based on data provided in the literature [20]. Moreover, DSC analysis reveals other characteristic temperatures such as glass transition (*T_g_*), melting (*T_m_*), and cold-crystallisation temperature (*T_cc_*).

### 2.6. Bacillus Amyloliquefaciens Biomass Designation

The biomass of bacterial cells in culture with the addition of the PLAG and PLAP tested carriers was determined using the weight method. The carriers prepared according to the procedure described in one of the previous articles written by the author [39] were inoculated with an indigenous bacterial strain *Bacillus amyloliquefaciens* isolated from the digested sewage sludge. The inoculated samples (except for control samples) were incubated at the temperature of 24 °C for 5 days (in the Compact Shaker KS 15 B incubator made by Edmund Bühler GmbH, Bodelshausen, Germany). After 5 days, the cultures, including the controls, were centrifuged (at 15,000 rpm for 15 min, in the Universal 16 R centrifuge made by Hettich Kirchlengern, Germany). The biomass of bacterial cells, grown on the substrate along with the carriers, was determined as the difference in weight (g) of the uninoculated substrate and the substrate inoculated with an autochthonous strain of bacteria *Bacillus amyloliquefaciens*.

### 2.7. Analysis of the Biochemical Activity of the Digestate

The digestate samples underwent biochemical analysis by means of the spectrophotometric method and dehydrogenase activity (DHA) that was measured using the method developed by Camiña et al. (1998) [42], with some modifications. Samples of approximately 5 mL were incubated at 30 °C, at a pH of 7.4 for 24 h with 2,3,5-triphenyltetrazolium chloride (TTC). Triphenylformazan (TPF) was yielded, extracted with 96% ethanol, and measured using a spectrophotometer at 285 nm. The dehydrogenase activity was expressed as µmol TPF mL^−1^ DM of digestate 24 h^−1^.

### 2.8. Statistical Analysis

Statistical analyses were performed with Statistica 13.3 software (StatSoft Inc. 2013, Tulsa, OK, USA). Two-way ANOVA was applied to determine the significance of the variation in the enzymatic activity. Tukey’s test was performed to calculate homogeneous mean subsets at a level of significance of *p* < 0.05. Principal component analysis (PCA) was performed using Past 3.25 software (Oslo, Norway).

## 3. Results and Discussion

### 3.1. Substrates and Inoculum Characterisation

The disposal of confectionery waste in anaerobic digestion is highly beneficial, due to its high BMP (biochemical methane potential), which is influenced by the chemical properties of that waste, including the high concentration of total solids (TS) and volatile solids (VS) (see Table 1). The carbon chains in carbohydrates contribute directly to the high C content (and the significant value of the C/N ratio) as a precursor of biomethane. A detailed discussion of the properties and advantages of using confectionery waste as substrates in the AD process has been included in the author’s earlier publications [3,5]. 

Dairy waste, including waste cheese (semi-skimmed cottage cheese), presents a slightly different chemical composition, distinguished by a lower content of total solids than is found in confectionery waste. However, the content of volatile solids is comparable, as is carbon content, resulting from the presence of carbohydrates (including lactose) and fat in its composition. Chemical biodegradation pathways for dairy materials have been analysed by the author and presented in her earlier article [37]. This paper [37], as well as other papers featuring the application of waste cheese [3], includes a detailed composition of the substrate and discusses the origin of nitrogen, including ammonium nitrogen, using, i.e., equations of casein decomposition. In dairy waste treatment by the AD process, the positive and negative effects of the elevated concentration of calcium and sodium macronutrients on the operation of an anaerobic reactor have been commonly indicated [43,44]. Excessive amounts of calcium may cause the formation of mill scale. However, for the amount that is dosed into the reactor in waste cheese in the co-digestion system (Table 2 in [3]), this macronutrient may promote the formation of cell aggregates. Potential problems arising from the slightly elevated sodium levels in waste cheese [37] and its acidic pH (Table 1), can be solved by using confectionery waste with complementary composition and buffering sludge in one system. The chemical basis for the transformation of sewage sludge into alkaline buffers for anaerobic biodegradation processes, based on nitrogen transformations, has been outlined by Pilarska et al. (2019) [33].

### 3.2. Polylactide Properties and Productivity

#### 3.2.1. Morphological and Microstructural Properties

**Figure 1:**
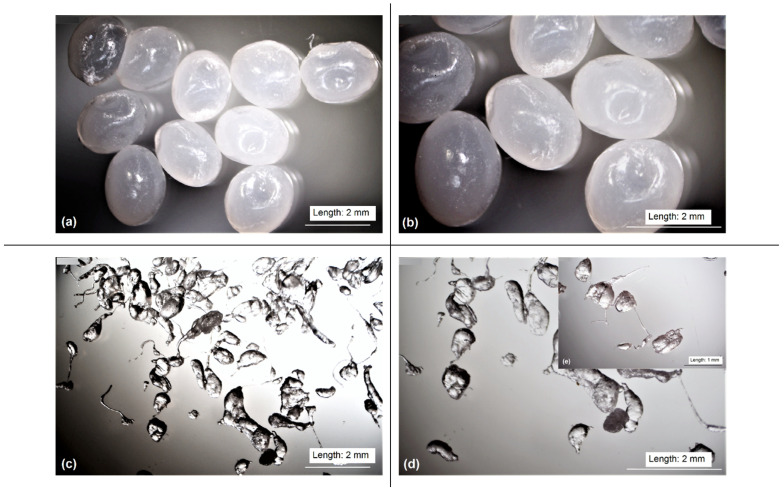
Microscopic pictures of PLA granules: (**a**)—×7 magnification, (**b**)—×10 magnification and PLA powder: (**c**)—×7 magnification, (**d**)—×10 magnification, (**e**)—×15 magnification.

The microscopic pictures (see Figure 1a–e) present morphological properties of PLA carriers produced and applied in the experiment in the form of granules and powder. These pictures provide some information on the granulate shape as well as on pulverised particles.

Granulates reveal regular, elliptic shapes with lengths in the range of 1.74 mm to 2.47 mm (Figure 1a,b). Quite different, irregular shapes can be assigned to the powdered particles that are seen in Figure 1c–e. In this case, the particles show a characteristic elongated shape with a long, thin thread. These kinds of threads are formed during the pulverising process as a consequence of plastic deformation of granulate and the temperature rise in the grinder. In order to complete the information on the morphology and compaction of particles in both forms of the material, their apparent density was measured. The value of the apparent density of PLA powder was 0.51 ± 0.39 g/cm^3^ and it was substantially lower than the density of granules, which was 0.75 ± 0.63 g/cm^3^. This confirms that after pulverisation the powder particles have an irregular shape and cannot be located so close to each other. Therefore, a lot of empty space should be found between those particles in the bulky state, which is the expected form. Therefore, the irregularity and non-uniform shapes of pulverised material contributed to their lower apparent density, which means that the bulky polylactide is not compact, and has a lot of empty space between the neighbouring particles.

**Figure 2:**
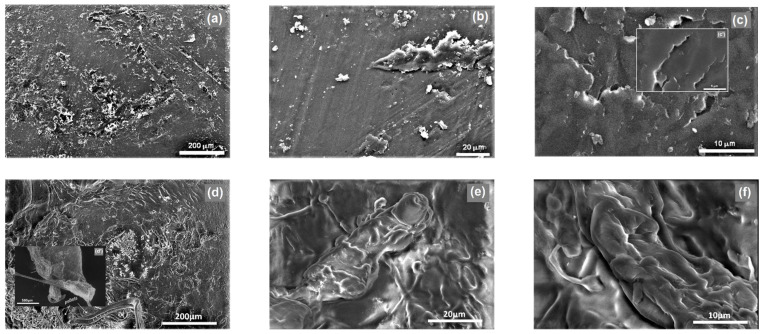
SEM micrographs of (**a**–**c**,**c’**)—PLA granules and (**d**,**d’**,**e**,**f**)—PLA powder, in different magnification.

The microstructural properties of PLA in the form of granules—PLAG (Figure 2a–c,c’) and powder—PLAP (Figure 2d,d’,e,f) have been assessed with the use of scanning electron microscopy (SEM). 

The images of the two forms of material, juxtaposed at different magnifications, highlight the changes that have occurred under the influence of the fragmentation of the granules performed in order to prepare a small fraction of the pulverised PLA. The surface of the PLAG sample was relatively smooth and clear, as shown in Figure 2c,c’, with shallow furrows that give a certain roughness to the granules, as can be seen in the image with the lowest magnification (Figure 2a). Similar SEM microscope images can also be seen in other publications [45]. Meanwhile, the mechanical processing of the granulated form of PLA resulted in the formation of an irregular microstructure, in which many undulations and coarse patches formed by particle agglomerates can be observed from the accompanying microscopic images. High stereoregularity in PLA chains boosts the formation of micropores inside the PLA films, producing neat PLA films with high porosity [23]. Images corresponding to the description presented above have also been presented in papers by other researchers [46,47], where changes to the PLA microstructure were often caused by chemical processing.

**Figure 3:**
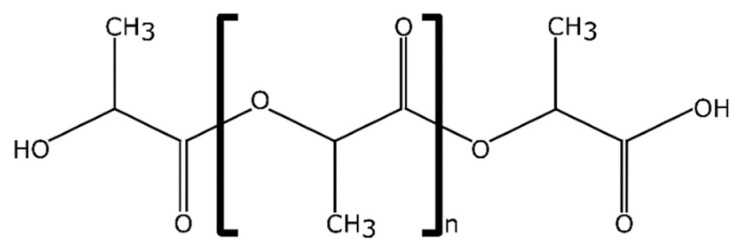
Structural formula of PLA.

Regardless of the form and morphological structure of the PLA carrier ((C_3_H_4_O_2_)_n_) applied in the process, its chemical composition remains unchanged. The elemental analysis of PLA, performed as part of this study, revealed a 50.15% share of carbon, 5.52% share of hydrogen, and 46.79% share of oxygen in its composition. As was confirmed by the SEM images (Figure 2), the mechanical processing applied affected the microstructure of the material and its tendency to form particle agglomerates (changes at the level of 10^−6^ m) but did not change the chemical composition of PLA (see Figure 3, Table 3). It is worth mentioning at this point that particles commonly used as building units in organic synthesis have dimensions ranging from a few to tens of angstroms (Å), which corresponds to 10^−10^ m [48].

The EDS (energy dispersive X-ray spectroscopy) technique was used to determine the chemical composition of PLA. The powdered form of the material, which is more convenient to use, was selected for the measurements. The analysis results presented in Table 3 confirm carbon’s highest weight content (56.34%) and atomic content (63.87%) in PLA and a slightly lower content (40.84% and 34.76%, respectively) of oxygen. In addition to these two elements, the presence of silica was also recorded, in the amount of 2.83% (*w/w*).

**Figure 4:**
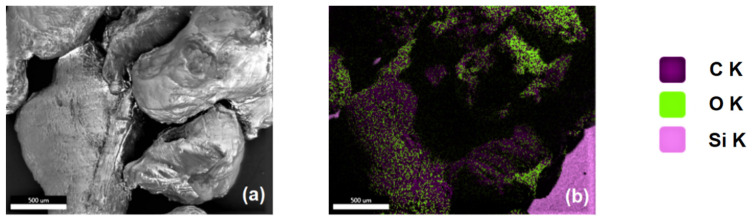

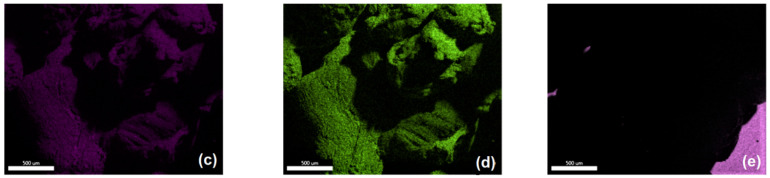
SEM-EDS images of PLA: (**a**)—SEM image of PLA and X-ray maps of (**b**)—carbon, oxygen, silicon; (**c**) carbon; (**d**)—oxygen; (**e**)—silicon.

The distribution of these elements in PLA was shown on EDS maps (Figure 4). The image, obtained using the SEM technique, confirmed the earlier observations of the irregular morphology of the PLA powder.

The X-ray Figure 4b map presents the distribution of three elements: C, O, and Si. The highest carbon densities were recorded in large areas of particle agglomerates (see Figure 4b). Observations in terms of the distribution of oxygen atoms (green) suggest that they are predominantly located on the peripheries of agglomerate structures. The visualisation presented in Figure 4c,d confirms the quantitatively comparable share of carbon and oxygen, while Figure 4e shows the small share of silica. Information on the local distributions of elements, especially carbon, is crucial for the use of PLA as a carrier and, at the same time, as a bacterial medium, under the conditions of the dissolution of the material in bioreactors or in the soil [45,49].

#### 3.2.2. Thermal Properties

The DSC analysis was applied to examine the thermal stability of the PLA biopolymer, which is crucial from the point of view of its various applications (including anaerobic digestion). This technique has made it possible to determine thermal changes during the heating of PLA in both states (PLAG and PLAP), and the temperatures accompanying it.

**Figure 5:**
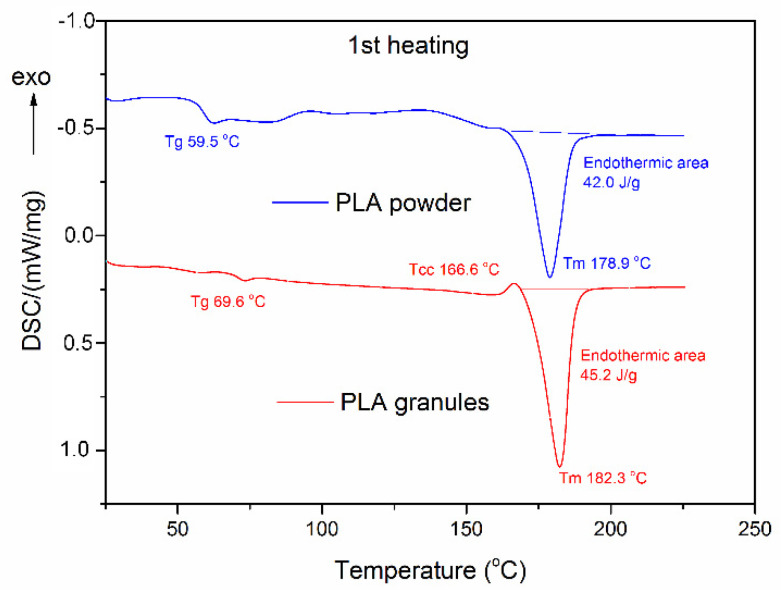
DSC traces of PLA in granular and powder states, obtained during the first heating scan.

Considering the DSC thermograms (blue curve—PLAP and red curve—PLAG) presented in Figure 5, there is no evidence of a recognisable recrystallisation effect during the first DSC scan, but the glass transition temperature for PLA powder is substantially lower than for PLA granules. This effect might be linked with the earlier pulverising operation and, as a consequence, with the increase in chain mobility due to the reduction in their length by means of mechanical processes.

**Figure 6:**
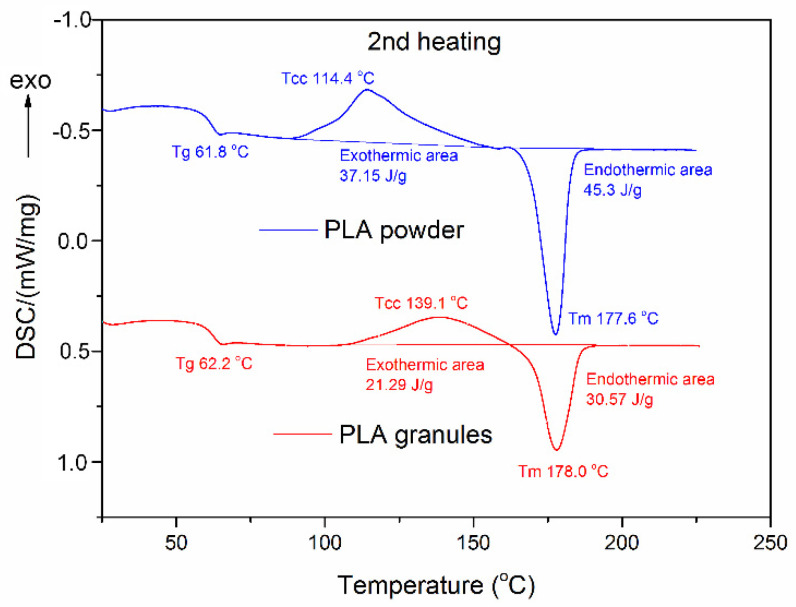
DSC traces of PLA in granular and powder states, obtained during the second heating scan.

During the second heating, the cold-crystallisation effect occurred for both samples (see Figure 6). In the case of the powdered state of PLA, the cold-crystallisation peak shifted to lower temperature (114.4 °C, see Table 4) compared to the original temperature for granules (139.1 °C). This shift could be also explained by the increase in chain mobility in the pulverised PLA. The cold-crystallisation effect is also much more significant in the case of PLAP, which, coincidentally, supports the assumption made above. Comparing the cold-crystallisation effects and the melting enthalpy for every tested sample, the final degree of crystallinity proved to be almost equal for PLA in the granular and powder state (see Figure 5 and Figure 6). In general, the temperature values obtained, including the glass transition temperature and melting temperature, agree with the data provided in the literature [45,50] and confirm that both forms of polylactide have thermal properties that enable its application as a cell carrier in mesophilic anaerobic digestion.

The values of key parameters, obtained on the basis of the DSC analysis performed for both carrier samples during the first and second heating scans have been collected in Table 4.

#### 3.2.3. Carrier Productivity

**Figure 7:**
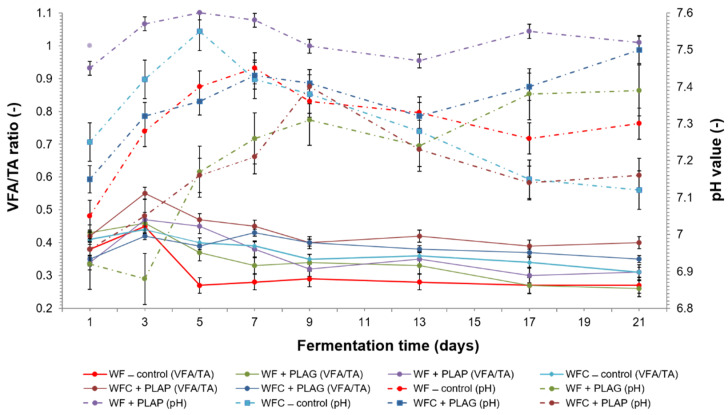
Changes in the pH and VFA/TA ratio during the anaerobic digestion of the batches.

The bacterial cell biomass in a culture of an indigenous strain of Gram-positive bacteria *Bacillus amyloliquefaciens* isolated from digested sewage sludge with the addition of the tested material was determined in this study (see Figure 7). 

Many *Bacillus* species offer significant benefits to biotechnology. This is because they are distinguished by fast growth, an efficient synthesis system, and the secretion of extracellular proteins [51]. Genome sequence analysis, presented by Daas et al. (2018) [52], identified the strain as *B. amyloliquefaciens* subspecies plantarum F11, and showed that the strain carries the gene clusters for the production of a number of bioactive and surface-active compounds.

In the test discussed, an increased proliferation of the isolate applied (*Bacillus amyloliquefaciens*) was recorded for the variant with the addition of PLAG (Figure S1a, Supplementary Materials). Cell biomass of 1.57 ± 0.09 g/100 mL was obtained after 5 days of cultivation. A much weaker colonising effect on the surface of the carrier was observed in the case of PLA, where the biomass of 0.52 ± 0.05 g/100 mL was obtained (Figure S1b, Supplementary Materials). This result was comparable to that obtained in the culture with lignin [41]. The productivity of cellular biomass in the test involving PLAG was three times higher than in the test featuring PLAP; it also exceeded the productivity achieved in the test featuring silica/lignin (1.12 ± 0.05 g/100 mL) [53], and diatomaceous earth/peat (9.35 ± 0.03 g/100 mL) [39]. 

The explanation for the aforementioned outcomes should be sought in the efficiency of immobilisation of cells on a given form of carrier, as well as the influence of physical and physicochemical phenomena occurring in the bioreactor. The immobilisation of microorganisms is based on their natural ability to adhere to surfaces, usually due to nutrient deficiency in the environment. The concentration of nutrients is slightly higher near the surface. Additionally, the intermolecular interactions near the surface are unbalanced, which contributes to a number of phenomena at the interface, including interfacial tension and adsorption. Cells adjacent to the surface have a metabolic advantage over cells freely suspended in solution [54]. Such a favourable location, in this case involving Bacillus amyloliquefaciens, was provided by a carrier in the form of PLA granules, which tended to float and occupy the near-surface zones during the experiment. In turn, porous and capillary surfaces, which occurred in the case of the powdered form of the carrier, as opposed to smooth, are characterised by poorer wettability [55]. Water, which is the habitat of microorganisms, is distinguished by high surface tension, while irregular surfaces and various nano-protrusions further decrease the contact area between a water droplet and the surface, causing the contact angles to increase considerably [56]. The obstructed contact with the aqueous environment certainly limited access to the medium in this study. It can also be assumed that micropores and free spaces in PLAP, which were not wetted with water, were filled with gases. The gaseous membrane, potentially formed at the interface, created a barrier between the cells and the matrix surface, which significantly impeded the immobilisation process. Moreover, during the experiment, it was observed that the powder had an unfavourable tendency to sink to the bottom of the bioreactor and stick the grains together, which further impaired the efficiency of the carrier. In such a situation, the low value of biomass in culture with PLAP is justified. The information obtained from the comparative analysis of the effect of PLA carriers on the proliferation of microorganisms provided a preview of anaerobic biodegradation results.

### 3.3. Anaerobic Digestion Stability and Performance

Apart from the chemical composition of the substrate, pH plays an important role in increasing the production rate and yield of VFA (volatile fatty acid) in anaerobic degradation. A pH range of less than 3 and more than 12, has been recognised on the basis of results of experiments conducted by other authors, to be inhibitory for acidogens [57]. However, the pH value in anaerobic digestion may vary within the range from 5.25 to 11, depending on the type of waste material. For example, for excess sludge, a pH as high as 10 is quoted as the optimum pH, while the optimum pH for food waste is around 7 [58].

In this study, pH was maintained between 6.92 (WF + PLAG) and 7.58 (WF + PLAP) in all samples and throughout the whole process (see Figure 7). This is a highly favourable result, given the risk of acidification in the case of confectionery fermentations at the first stage of the process. The upward trend in pH values, observed in virtually all samples, especially those with a protein substrate, was caused by the gradual removal of organic nitrogen from the raw material in the form of released ammoniacal nitrogen [3]. In the present study, ammonia was also the final product of the anaerobic digestion of protein (derived from cheese) at the hydrolysis stage [37]. 

**Figure 8:**
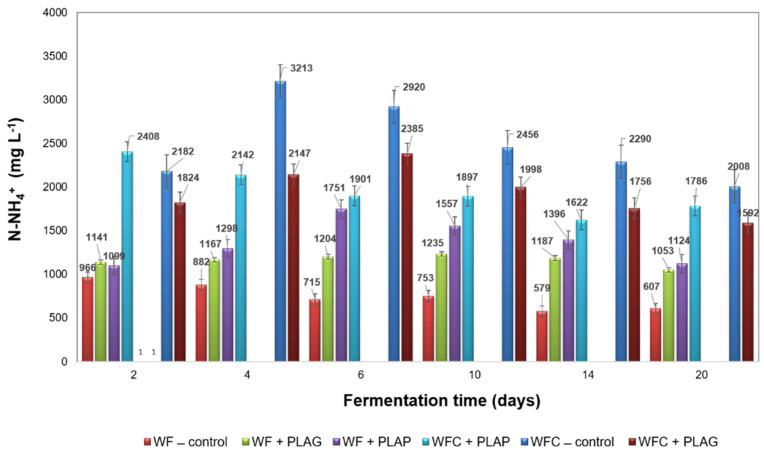
Changes in N-NH_4_^+^ during anaerobic digestion of the batches.

After approximately 4–6 days of the AD process an increase in the concentration of N–NH_4_^+^, to as much as 3213 mg L^−1^ (Figure 8), was observed for WFC—control, which alkalised the system slightly to pH 7.55 (Figure 7). Thus, the total ammonium nitrate (TAN) can act as a buffer in the digested system, providing partial alkalinity and preventing acidification, but also, according to the literature, it can also act as a nutrient for anaerobic microorganisms [58]. On the other hand, as the concentration of N-NH_4_^+^ increases, there can be a reduction in the enzymatic activity of microorganisms (and an inhibition of the process) [43], which, however, did not pose a risk to the values obtained in the study.

Therefore, the selection of carbohydrate and protein food-based materials as co-substrates is beneficial, as has been confirmed in the author’s earlier publications [39,53]. Meanwhile, combining such a selection of substrates with a stabilised sewage sludge as inoculum guarantees a stable implementation of the process and its high efficiency [3].

The chemical basis of the buffering properties of digested sewage sludge has been discussed in detail in earlier publications by Pilarska et al. (2016, 2019) [33,37]. It is worth noting that sludge from wastewater treatment plants is a system with a complex composition and complex structure. The particles found in the sludge are distinguished by varied shapes and relatively small sizes. Due to their morphological properties, they have the ability to intensify surface phenomena, forming semi-suspensions that are referred to as permanent systems [36]. 

The stable course of matter decomposition in the experiment in question was also confirmed by VFA/TA, which is the second key parameter in the monitoring of the AD process (Figure 7). Its value, ranging from 0.27 (WF—control and WF + PLAG) to 0.55 (WFC + PLAP), points to some symptoms of destabilisation in the co-substrate system with the addition of PLA powder (values exceeding 0.4). From day 5 onwards, a gradual decrease in VFA/TA ratio was registered, which was associated with the decomposition and removal of organic matter. These results correlate with the values obtained for the chemical oxygen demand (COD_r_), as the drop in its value was observed from days 6–10, depending on the sample (see Figure S2, Supplementary Materials). Reducing the amount of oxygen consumed by oxidation reactions confirms the effective bioconversion and VS removal.

**Figure 9:**
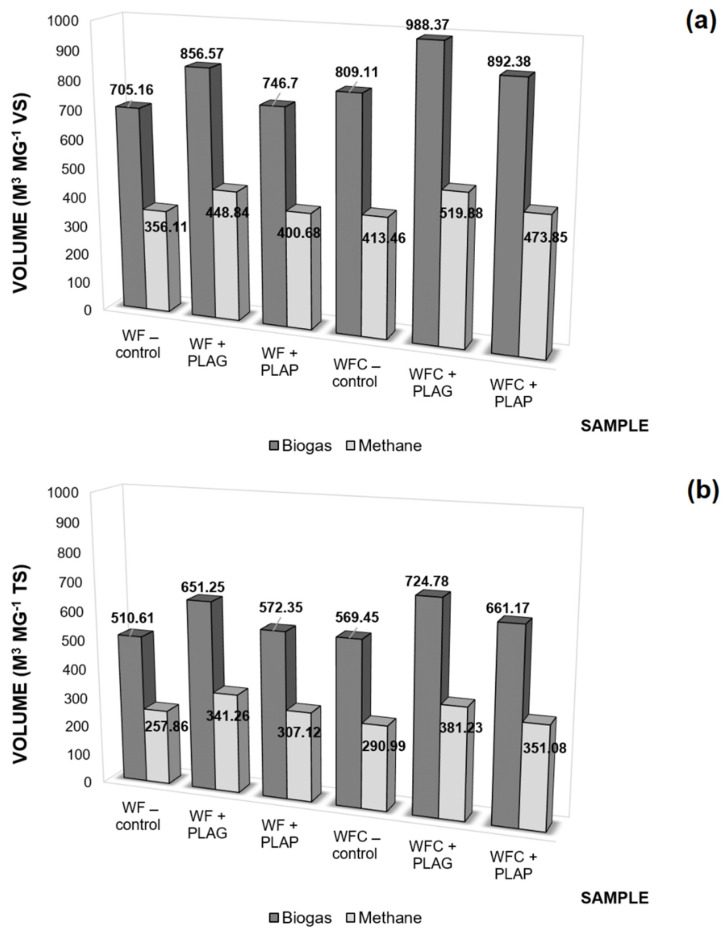
Biogas and methane efficiency from Mg of (**a**) volatile solids (VS) and (**b**) total solids (TS) obtained from the samples tested.

Analysing the BMP (biochemical methane potential) of the tested samples with one confectionery substrate, WF—control, and with the confectionery and protein co-substrate system, WFC—control (see Figure 9a), a favourable increase in the amount of biogas produced has been observed (from 705.16 m^3^ Mg^−1^ VS to 809.11 m^3^ Mg^−1^ VS), along with an increase in its calorific value—an increase in the content of CH_4_ from 356.11 m^3^ Mg^−1^ VS to 413.46 m^3^ Mg^−1^ VS. This conclusion was announced in the first study involving this co-substrate system [3]. The control results presented here provide a reference in establishing the effect of the application of PLAG and PLAP, as the carriers in AD. The registered amounts of biogas, including methane, after conversion to volatile solids, and with total solids (Figure 9a,b) indicate a decisive improvement in efficiency as a result of the addition of PLA granules. Compared to control samples, this means an increase in the methane produced by approx. 26%, both in relation to WF—control (from 356.11 m^3^ Mg^−1^ VS to 448.84 m^3^ Mg^−1^ VS), and in relation to WFC—control (from 413.46 m^3^ Mg^−1^ VS to 519.98 m^3^ Mg^−1^ VS). A lower yield of gas was obtained in the case of the application of PLA powder, as these results are comparable to (WF—control vs. WF + PLAP) and slightly higher (WFC—control vs. WFC + PLAP) than the control results. These results were predicted by the test presented earlier (Section 3.2.2) using an indigenous strain of *Bacillus amyloliquefaciens* bacteria. The positive effect of PLAG on cells is attributed to the effective immobilisation of cells on the surface of this carrier and better wettability of the nearly smooth surfaces of the granules. The good adhesion to the surface of the material and the ease of migration into the near-surface areas improved the access of cells to the medium, resulting in their increased proliferation and elevated enzymatic activity, as demonstrated by the test results described in Section 3.4. In turn, the factors determining a different situation in the reactor with PLAP addition were explained in Section 3.2.2. 

It is interesting to note that the addition of granulated PLA had a slightly more favourable impact on improving the efficiency of the AD process in the systems discussed than the carrier materials researched by Pilarska et al. earlier [7,39,53]. In principle, the effect of PLAG is comparable to the silica/lignin system (S/L; 4:1), [53] while the introduction of powdered PLA resulted in the lowest efficiency among the tested carriers. It was lower than in the diatomaceous earth/peat (DEP; 3:1) system [39]. The efficiencies of the AD process, obtained for WAF + PLAG are comparable to the BMP of high-calorific fatty waste [33,59].

At the same time, the material balance calculations performed showed that the WFC + PLAG system achieved the highest biomass conversion level (0.9884). A slightly lower level of 0.95064 was noted for WFC + S/L, while the lowest level was achieved by the control systems with the addition of PLAP, particularly without co-substrate (0.7467 for WF + PLAP). High biomass conversion rates depend on many factors, including, above all, the quantitative and qualitative composition of the digestion mixture, the presence of inhibitors, parameters of the process, and the condition of the bacterial microbiome [7,60].

### 3.4. Dehydrogenase Activity

The activity of dehydrogenase may serve as an indicator of microbial activity of the active sludge [61] and, thus, as one of the more important parameters of anaerobic digestion monitoring. The activity of dehydrogenase depends on the type of microorganisms, the phase of their growth, and the physicochemical composition of the digested suspension. 

**Figure 10:**
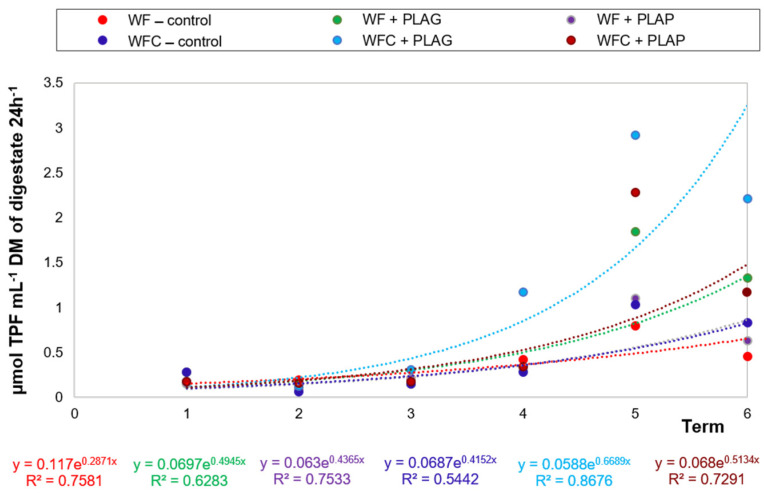
Dehydrogenase activity changes found in the digested samples. Explanation: The same letter indicates a lack of significant differences (*p* < 0.05).

Statistical analysis confirmed that the activity of dehydrogenase in food waste subject to digestion showed statistically significant variation in response to the sampling date and the type of the experimental variant (see Figure 10). As the digestion progressed, the activity of DHA grew steadily in the tested objects, reaching the highest level on the 5th digestion date (18th day), including the clearly dominant level in the WFC + PLAG variant (blue curve). After the 5th digestion date (up to the 20th day of the process), the enzyme activity diminished in each of the tested samples, which was due to the bioconversion to methane and carbon dioxide, and which implied the loss of nutrient solution. The lowest level of activity of the enzymes, with the exception of WF—control, was observed on the 2nd date (4th day) of the process (red curve). 

Biochemical exploration of the digested waste confirmed that adding protein waste to the co-substrate, and the addition and form of the carrier both had a significant impact on DHA. A higher level of dehydrogenase activity was observed in the variants with the addition of cheese and with the addition of the PLAG, which had a direct impact on the yield of methane in these particular samples (Figure 10).

**Figure 11:**
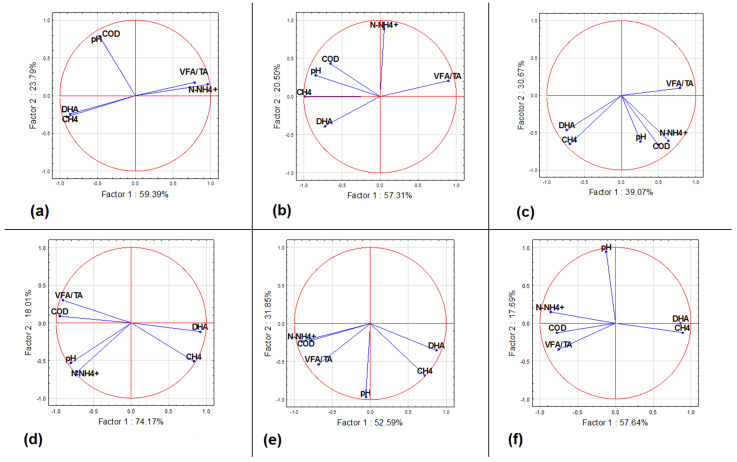
Distribution of dehydrogenase activity as well as chemical properties in the tested samples: (**a**) WF—control; (**b**) WF + PLAG; (**c**) WF + PLAP; (**d**) WFC—control; (**e**) WFC + PLAG; (**f**) WFC + PLAP, in two PCA axes. Legend: DHA—dehydrogenase activity, VFA/TA ratio—volatile fatty acids-to-total alkalinity ratio, N-NH_4_^+^—ammonium nitrate, COD—chemical oxygen demand.

Meanwhile, principal component analysis (PCA) confirmed the significant impact of the experimental variant on the type of relationship between the tested chemical parameters and DHA activity in the digested samples (Figure 11). A strong relationship was demonstrated between the level of dehydrogenase activity and methane emissions in all objects, particularly in WF + PLAG (*r* = 0.8579). No significant impact of the pH value on DHA activity has been observed: pH remained essentially unchanged, with a slight upward trend in selected variants (see Figure 7). Moreover, a negative correlation between the activity of the tested enzymes and the content of ammonium nitrogen ions was demonstrated in all sites. The content of N-NH_4_^+^ in the digested waste (Figure 11) proved to be an important predictor of dehydrogenase activity. Summing up, the addition of cheese as a source of fat and amino acids [37] was, in addition to the PLAG carrier, a factor determining the efficiency of methane generation. 

## 4. Conclusions

The results of the analysis of physicochemical properties of carriers, including the key DSC thermal analysis and the results of enzymatic activity of the samples, and the amount of *Bacillus amyloliquefaciens* cell biomass obtained in the culture with the addition of the tested PLA granules and PLA powder, confirmed that PLA can be an effective cell carrier in mesophilic AD. The addition of PLAG delivered better results of bacterial proliferation than the addition of PLAP, which was explained by the effective immobilisation of cells on the PLAG surface, better wettability of the nearly smooth surfaces of the granules and easier access to the nutrient solution. The increase in the volume of the generated methane observed for samples with the addition of PLAG equalled approximately 26%: for waste wafers, it increased from 356.11 m^3^ Mg^−1^ VS (WF—control) to 448.84 m^3^ Mg^−1^ VS (WF + PLAG), while for the co-substrate system it went up from 413.46 m^3^ Mg^−1^ VS, (WFC—control) to 519.98 m^3^ Mg^−1^ VS (WFC + PLAG). At the same time, the WFC + PLAG system was distinguished by the highest levels of enzymatic activity, which had a direct influence on the more effective bioconversion of substrates and efficiency of the process. The dynamic of changes in the AD monitoring parameter values confirmed the stable and correct nature of the process. 

Meanwhile, principal component analysis determined that the N-NH_4_^+^ released during the process is an important predictor of dehydrogenase activity, which could play the role of buffer in the system and of a nutrient for microorganisms in the study conducted. It was concluded that combining carbohydrate and protein co-substrates with PLA granules being a cell carrier in mesophilic AD guarantees high efficiency of methane production and may constitute an alternative method of management of waste PLA.

## Figures and Tables

**Table 1:** Physicochemical properties of substrates and inoculum.

Materials	pH	Cond.	TS	VS	C/N Ratio	C	N	N-NH_4_
	-	(mS cm^−1^)	(wt %)	(wt %_TS_)	-	(wt %_TS_)	(wt %_TS_)	(wt %_TS_)
Waste wafers	7.12	2.24	74.85	98.24	54.83	45.51	0.83	0.25
Waste cheese	5.38	68.55	42.05	91.36	4.25	47.78	11.24	0.39
Inoculum	6.93	29.87	2.96	73.29	3.04	24.42	8.02	4.12

Cond.—conductivity, TS—total solids, VS—volatile solids.

**Table 2:** Composition and selected properties of the substrate/inoculum batches.

Batches	WF (g)	CE (g)	Carrier (g)	Inoculum (g)	pH	TS (%)	VS (%_TS_)
WF − control	9.80	-	-	830.0	7.05	3.86	75.78
WF + PLAG	9.80	-	20.0	830.0	7.13	3.84	76.03
WF + PLAP	9.80	-	20.0	830.0	7.08	3.98	76.64
WFC − control	5.50	2.90	-	832.0	6.96	3.47	72.93
WFC + PLAP	5.50	2.90	20.0	832.0	6.85	3.58	74.09
WFC + PLAG	5.50	2.90	20.0	832.0	6.92	3.37	72.74

**Table 3:** Chemical composition of PLA.

Element	Weight (%)	Atomic (%)	Error (%)	Net Intensity
C K	56.34	63.87	12.68	2983.87
O K	40.84	34.76	17.47	1924.51
Si K	2.82	1.37	2.56	308.03

C—carbon; O—oxygen; Si—silicon; K—shell X-ray emission.

**Table 4:** Summary of DSC results obtained during two scans of every sample.

Carriers		First Scan		Second Scan					
	T_g1_ (°C)	T_m1_ (°C)	∆H_m1_ (J/g)	T_g2_ (°C)	T_cc_ (°C)	∆H_cc_ (J/g)	T_m2_ (°C)	∆H_m2_ (J/g)	X_c2_ (%)
PLA granules	69.6	182.3	45.2	62.2	139.1	21.29	178.0	30.57	9.9
PLA powder	59.5	178.9	42.0	61.8	114.4	37.15	177.6	45.30	8.7

T_g1_, T_g2_—glass transition temperature at 1st and 2nd heating; T_m1_, T_m2—_melting peak temperature at 1st and 2nd heating; T_cc_—cold-crystallisation peak temperature at 2nd heating; X_c2_—crystallinity at 2nd heating; ∆H_m1_, ∆H_m2_—enthalpy of melting at 1st and 2nd heating, ∆H_cc_—enthalpy of cold-crystallisation at 2nd heating.

## Supplementary Materials

The following supporting information can be downloaded at: https://www.mdpi.com/article/10.3390/ma15228113/s1, Figure S1. *Bacillus amyloliquefaciens* culture with added carriers: (a) PLA granules and (b) PLA powder. Figure S2. Changes in COD_Cr_ during anaerobic digestion of the batches.
