# Supplementary material for: Polylactide (PLA) as a Cell Carrier in Mesophilic Anaerobic Digestion—A New Strategy in the Management of PLA
Source: Materials (Basel). 2022 Nov 16;15(22):8113. doi: 10.3390/ma15228113 (PMC9697477; doi:10.3390/ma15228113)
Supplement: Supplementary file 1 [file materials-15-08113-s001.zip › materials-2008688-supplementary.pdf]

materials

MDPI

Supplementary Materials

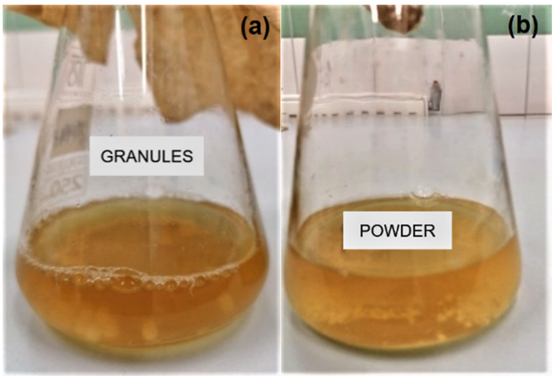

Figure S1. *Bacillus amyloliquefaciens* culture with added carriers: (**a**) PLA granules and (**b**) PLA powder.

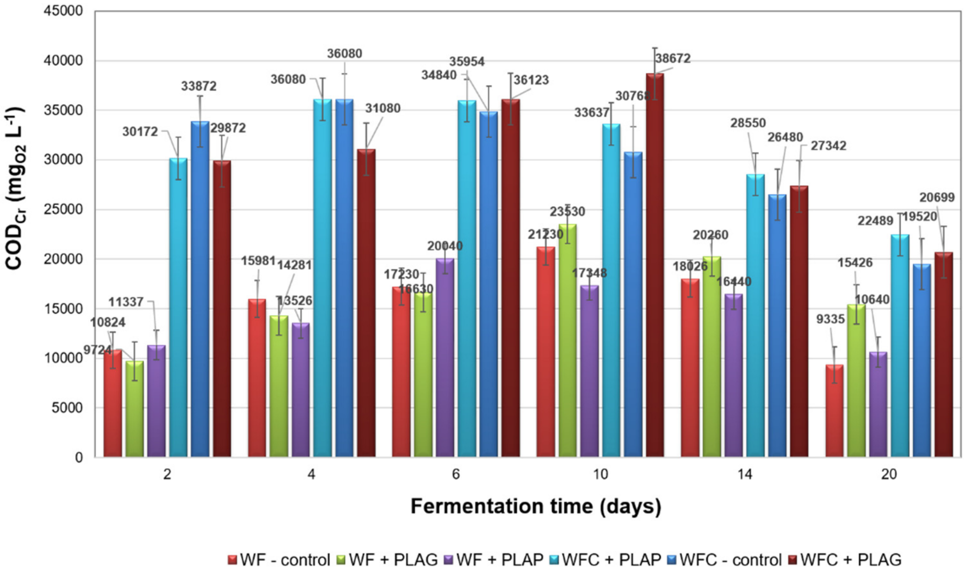

Figure S2. Changes in COD_{Cr} during anaerobic digestion of the batches.
